# Supplementary material for: Content-rich biological network constructed by mining PubMed abstracts
Source: BMC Bioinformatics. 2004 Oct 8;5:147. doi: 10.1186/1471-2105-5-147 (PMC528731; doi:10.1186/1471-2105-5-147)
Supplement: Additional File 2 — The original results of the above study (non-essential files are deleted to keep the file size under the limit set by BMC bioinformatics). [file 1471-2105-5-147-S2.bz2 › chilibotAdditionalFile2/dip30/407ID11468284E10068/html/CSF2_CSF3R.html]

 


 **CSF2** and **CSF3R** 
  
Found 185 abstracts in PubMed, retrieved 30.  
 

 What does Google say? 
 PDF only 
| .edu only 

---

**Interactive relationship** (e.g. stimulation, inhibition, etc)

**Stimulatory relationship**- Granulocyte colony stimulating factor  [ **CSF2** ]  G CSF induces proliferation, maturation, and functional activities of myeloid progenitors and mature neutrophils through a specific receptor, the G CSF R  [ **CSF3R** ] .  Ref: 12586631 Blood, 2003
**Neutral relationship**

**Non-interactive relationship** (e.g. studied together, co-existance, homology, etc.)

- To elucidate the function of Cb2 in myeloid leukemia cells, a set of in vitro experiments was carried out using 32D G CSF R  [ **CSF3R** ]  granulocyte colony stimulating factor  [ **CSF2** ]  receptor cells transfected with exon 1B exon 2 Cb2 complementary DNA and a myeloid cell line carrying a virus insertion in Cb2 ie, NFS 78.  Ref: 11929767 Blood, 2002
